# Hysteroscopic injections of autologous endometrial cells and platelet-rich plasma in patients with thin endometrium: a pilot randomized study

**DOI:** 10.1038/s41598-023-27982-w

**Published:** 2023-01-18

**Authors:** Zulfiia Efendieva, Polina Vishnyakova, Inna Apolikhina, Daria Artemova, Kirill Butov, Elena Kalinina, Tatiana Fedorova, Anna Tregubova, Aleksandra Asaturova, Timur Fatkhudinov, Gennady Sukhikh

**Affiliations:** 1 National Medical Research Center for Obstetrics, Gynecology and Perinatology Named After Academician V.I. Kulakov of Ministry of Healthcare of Russian Federation, Moscow, Russia; 2 Peoples’ Friendship University of Russia (RUDN), Moscow, Russia; 3 I.M. Sechenov First MSMU of Ministry of Health of Russia (Sechenov University), Moscow, Russia; 4 Avtsyn Research Institute of Human Morphology of Federal State Budgetary Scientific Institution “Petrovsky National Research Centre of Surgery”, Moscow, Russia; 5 Oncology and Immunology, Dmitry Rogachev National Medical Research Center of Pediatric Hematology, Moscow, Russia

**Keywords:** Infertility, Phase II trials

## Abstract

The aim of this study was to evaluate the efficacy of hysteroscopically controlled injections of autologous platelet-rich plasma (PRP) and autologous endometrial cells as a treatment for infertile women with thin endometrium. The study enrolled 115 patients with thin endometrium (< 7 mm at implantation window) and infertility, who were divided into groups: Group 1 (the control) underwent conservative therapy; Group 2 received intraendometrial PRP injections instead of the conservative therapy; Group 3 received identical injections after conservative therapy; Group 4 received injections of the autologous endometrial cells suspended in PRP. A single injection dose of PRP contained 0.6–0.7 × 10^11^ of platelets. The levels of PDGF-BB and VEGF in PRP were increased compared with ordinary plasma. The autologous endometrial cells, obtained from pipelle biopsies, constituted heterogeneous cell populations containing stromal and epithelial cells. Intraendometrial PRP injections had significant impact on endometrial thickness and local microcirculation in Group 2 and Group 3. In Group 4, injections of PRP reinforced with endometrial cells also facilitated a significant increase in endometrial thickness. This work describes a novel approach for infertility treatment in patients with refractory thin endometrium. PRP injections and injections of the endometrial cells suspended in PRP into endometrium enhanced cell proliferation and angiogenesis.

## Introduction

The uterine factor infertility remains unresolved issue. Under-thickened endometrium (< 7 mm at implantation window) has been associated with reduced implantation rates and poor pregnancy outcomes in the assisted reproductive technology (ART) programs. Thin endometrium indicates the unresponsiveness to growth stimuli (refractory state) and represents an important clinical indicator for cancellations of embryo transfer (ET) cycles^1–3^.

Administration of autologous platelet-rich plasma (PRP) to the uterine cavity is beneficial for endometrial thickness and has been shown to improve the rates of implantation and clinical pregnancy achieved in ART cycles compared with hormone replacement alone^4^. Autologous PRP is known to stimulate cell growth and angiogenesis^5,6^; for instance, it promotes migration and adhesion of endometrial stromal cells in vitro^7^. The mechanism involves growth factors contained in platelet granules and released upon platelet activation. Despite the benefits of the minimally-invasive infusions of PRP into uterine cavity^4,6^, such interventions (termed 'instillations') fail to deliver high concentrations of growth factors to the basal layer of endometrium, especially with their volumes limited to 1–2 mL.

Other treatment options for refractory thin endometrium involve progenitor/stem cells. In healthy endometrium, cells of the basal layer are enormously proliferative – their self-renewal ensures reconstitution of the functional layer in every menstrual cycle^8^. An important strategy of stimulating endometrial growth involves bone marrow-derived stem cells^9^. Particularly promising results were achieved with infusions of autologous CD133 + bone marrow-derived mesenchymal stromal/stem cells (MSCs) into uterine arteries^10^. High therapeutic potential of eutopic cells in combination with autologous PRP for the treatment of endometrial insufficiency was demonstrated in clinical studies^11^. Intraendometrial injections of the endometrium-derived autologous MSCs and autologous PRP in patients with refractory thin endometrium significantly increased endometrial thickness and improved clinical pregnancy rates. Of 29 patients subjected to the treatment followed by ET under hormone replacement, 23 (79%) entered clinical pregnancies concluded with live births in 10 cases (34.5%)^11^; of note, the curative cell product was entirely stromal.

**Figure 1:**
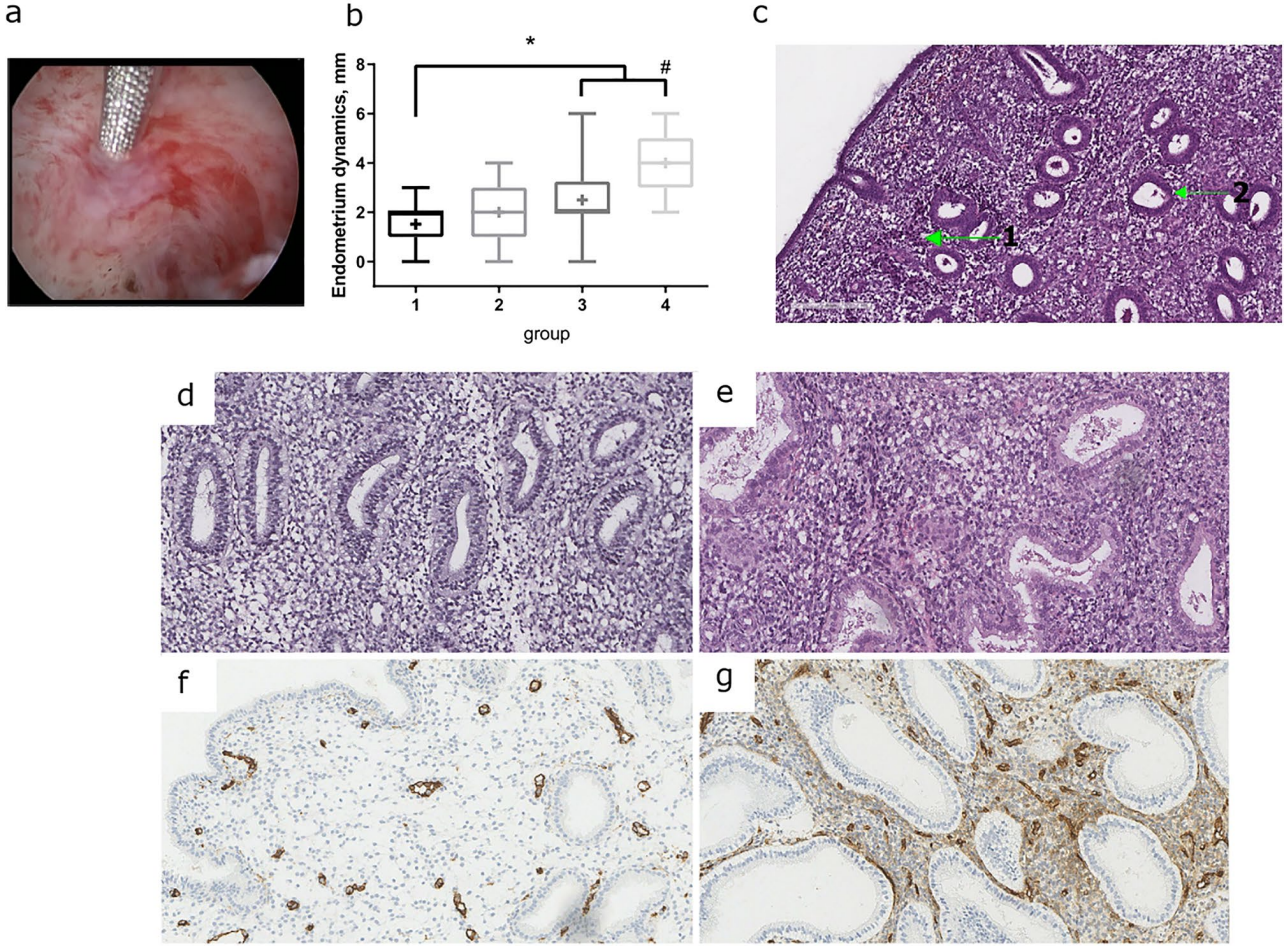
Endometrium parameters. Injection of autologous PRP – the image shows endoscopic needle inserted in the endometrium to the controlled depth of 2–3 mm (**a**). Endometrium thickness dynamics for the groups (**b**): * – *p *< 0,05; # – *p *< 0,05 for Group 4 vs Group 2. Histological image of endometrium with signs of fibrosis and atrophy (**c**): **1**, fibrotic foci in the stroma adjacent to endometrial glands; **2**, atrophic gland; hematoxylin and eosin, magnification × 100. Endometrium of an early stage of secretion in a patient who became pregnant ((**d**) – staining with hematoxylin and eosin, (**f**) – immunohistochemical expression of CD34); endometrium of an inferior secretion phase in a patient who did not become pregnant ((**e**) – staining with hematoxylin and eosin, (**g**) – immunohistochemical expression of CD34). Magnification × 200. Software: ImageScope RRID:SCR_014311.

In this study, we used an alternative protocol for PRP delivery to the site of action. The enhanced efficacy was anticipated on the basis of (1) the larger injection volume (up to 40 mL) and (2) the hysteroscopic image-controlled precision delivery of growth factors to the basal layer of endometrium. This pilot study also evaluates the efficacy of intraendometrial injections for autologous PRP in pure form or mixed with the minimally manipulated endometrial cells (comprising both epithelial and stromal components) as compared with conservative management (physiotherapy) in patients with refractory thin endometrium (Fig. 1a).

## Results

### Patient data

The age of the patients did not differ significantly between the groups and averaged 34.9 ± 4.0 years. The body mass index was 22.4 ± 3.4 kg/m^2^. The cohort was uniform in terms of luteinizing hormone and follicle-stimulating hormone profiles, blood levels of estradiol and somatic medical history. Other indicators (age at menarche, menstrual cycle length, period duration) were similar as well, and comparable proportions of patients in each group presented with scanty menstrual bleedings.

Most of the patients had a history of repeated intrauterine interventions, including hysteroscopies with dilation and curettage (D&C) of uterine cavity and cervical canal, endometrial polyp resections and diagnostic laparoscopies. The incidence of births, ectopic pregnancies and abortions was similar. There were no losses and exclusions in any group after randomization. The data for Groups 1–3 is given in Table 1 (for this type of analysis Group 4 was excluded for the sake of statistical accuracy due to its small size, n = 5).

**Table 1:** Clinical history indicators for Groups 1–3.

Events and conditions	Group 1 (n = 30)	Group 2 (n = 42)	Group 3 (n = 38)	p-value
Age, years*	33.9 ± 3.7	35.1 ± 4.2	35.4 ± 4.1	0.2911
Hypomenorrhea**	19 (63%)	29 (69%)	24 (63%)	0.8
Live birth**	7 (23%)	6 (14%)	9 (24%)	0.5
Pregnancy loss**	10 (33%)	23 (55%)	24 (63%)	**0.045**
Chronic endometritis**	21 (71%)	36 (87%)	34 (89%)	0.08
Hysteroscopic resection of endometrial polyps **	19 (63%)	26 (62%)	16 (42%)	0.12
Diagnostic hysteroscopy, curettage**	27 (90%)	32 (76%)	28 (74%)	0.2
Curettage after pregnancy loss**	9 (30%)	22 (52%)	24 (63%)	**0.013**
Hysteroresectoscopy, dissection of synechiae **	4 (13%)	11 (26%)	11 (29%)	0.3
History of IVF implantation failure and ET cancellations**	25 (83%)	31 (74%)	34 (89%)	0.4

*the data are presented as mean ± SD, subject to ANOVA.

**the data are presented as raw counts and % incidence, subject to exact Fisher test.

Significant values are in bold.

It should be noted that, despite the lack of formal statistical assessment, Group 4 had clinical history loads comparable with the rest of the cohort. IVF implantation failures and ET cancellations were encountered by 4 patients out of 5, and 4 patients out of 5 underwent diagnostic hysteroscopies. All patients in this group (n = 5) had at least one D&C procedure in anamnesis.

Prior to the enrollment, the vast majority of the cohort had had a history of IVF implantation failure and/or cryotransfer cycle cancellation due to endometrial growth insufficiency. The infertility duration was comparable in all groups and averaged 4.7 ± 3.2 years. All patients had previously received estrogen therapy for thin endometrium, administered orally or transdermally in high doses, as well as repeated courses of physiotherapy. It is important to note that, in addition to hormonal therapy, the patients had also received auxiliary ('add-on') interventions, including non-invasive intrauterine infusions (instillations) of G-CSF and autologous PRP (0.5–1 mL).

### Conventional ultrasonography

Endometrial thickness measured during the estimated implantation window (< 7 mm in all patients according to the inclusion criteria, see *Enrollment*) was statistically similar in all groups, constituting 5.9 ± 1.2 mm in Group 1 (n = 30), 5.3 ± 1.5 mm in Group 2 (n = 42), 5.4 ± 1.4 mm in Group 3 (n = 38) and 5.6 ± 0.5 mm in Group 4 (n = 5). The scans revealed a significant increase in endometrial thickness at implantation window of the next menstrual cycle in all Groups (*p* < 0.00001 for Groups 1, 2 and 3; *p* = 0.0431 for Group 4). The dynamics are specified in Table 2.

**Table 2:** Ultrasonography measurements of endometrial thickness before and after the therapy.

Group	Before therapy, mm	After therapy, mm	Gain, mm
1 (n = 30)	5.9 ± 1.2	7.4 ± 1.2	1.5 ± 0.6 (0;3)
2 (n = 42)	5.3 ± 1.5	7.4 ± 1.8	2 ± 1.2 (0;4)
3 (n = 38)	5.4 ± 1.4	7.9 ± 1.6	2.5 ± 1.3(0;6)
4 (n = 5)	5.6 ± 0.5	9.6 ± 1.1	4 ± 1.4(2;6)

The data are presented as mean ± SD (min;max).

The gain in endometrial thickness in Group 3 was significantly higher than in Group 1 (*p* = 0.003). In Group 4, (n = 5), the gain was significantly higher compared with both Group 1 (*p* = 0.005) and Group 2 (*p* = 0.037), whereas the corresponding difference between Groups 3 and 4 was below the level of significance (*p* = 0.177) (Fig. 1b).

### Color Doppler recording and Doppler spectrogram ultrasonography

The rates of visualization of the uterine spiral arteries for Groups 2 and 3 were significantly higher after the treatment than before (respectively, *p* = 0.0002 and *p* = 0.0005), whereas corresponding rates for Group 1 were statistically similar (no dynamics; Table 3). In Group 4 (n = 5), spiral arteries were detectable in 2 patients before the treatment and in all patients after the treatment.

**Table 3:** Success ratios of Doppler-assisted visualization of uterine spiral arteries before and after the therapy.

Group	Before therapy	After therapy	p-value
1 (n = 30)	17 (56.6%)	22 (73.3%)	0.0625
2 (n = 42)	18 (42.8%)	31 (73.8%)	0.0002
3 (n = 38)	16 (42.1%)	28 (73.6%)	0.0005

The data are presented as raw counts and % rates, subject to McNemar’s binomial test.

A relationship between endometrial thickness and local microcirculatory hemodynamics was specified statistically. Significant associations of endometrial thickness parameters with angle-independent Doppler indices were revealed, including negative (inverse) correlations between M-mode echo and pulsatility index (*r*_*s*_ = − 0.5; *p* < 0.05), endometrial thickness and resistance index (*r*_*s*_ = − 0.52; *p* < 0.05), and M-mode echo and systolic-diastolic velocity ratio (*r*_*s*_ = − 0.53; *p* < 0.05) for uterine spiral arteries.

### Histological assessment

Of the histological specimens (n = 25), 18 corresponded to the early and middle secretory phase. The rest 7 specimens showed secretory phase insufficiency with sparse deformed endometrial glands, signs of atrophy and fibrotic foci (Fig. 1c), as well as vascular depletion, characteristic of patients with under-thickened endometrium (the vessels were mainly represented by small spiral arteries and thin-walled venules). The expression indices of the studied immunohistochemical markers (PGR, ESRRA, LIF, CD34) are presented in Table 4, as well as in Fig. 1d–g. Statistically significant differences in the level of CD34 expression (*p *= 0.035) were found in the groups of patients with pregnancy compared with patients who are unable to get pregnant.

**Table 4:** Expression of estrogen (ESRRA) and progesterone (PGR) receptors and their ratios, leukemia-inhibiting factor (LIF) and CD34 in the endometrium of patients with pregnancy and patients who are unable to get pregnant.

Indicators	Patients with pregnancy		Patients who are unable to get pregnant		p-value
	Me	Q1-Q3	Me	Q1-Q3	
PGR in glands	300.0	267.5–300.0	300.0	180.0–300.0	0.437
PGR stromal	295.5	289.5–297.0	297.0	294.0–300.0	0.247
ESRRA in glands	300.0	260.0–300.0	297.0	230.0–300.0	0.611
ESRRA stromal	294.0	240.0–297.5	294.0	260.0–300.0	1
PGR/ESRRA in glands	1.00	1.00–1.04	1.00	0.86–1.00	0.225
PGR/ESRRA stromal	1.01	1.00–1.22	1.02	1.00–1.13	0.611
LIF	295.5	233.5–298.5	270.0	190.0–294.0	0.295
CD34	3.0	2.0–4.0	4.0	3.0–4.0	**0.035**

Significant values are in bold.

### Immunochemical characterization of PRP

#### Platelet granule content

**Figure 2:**
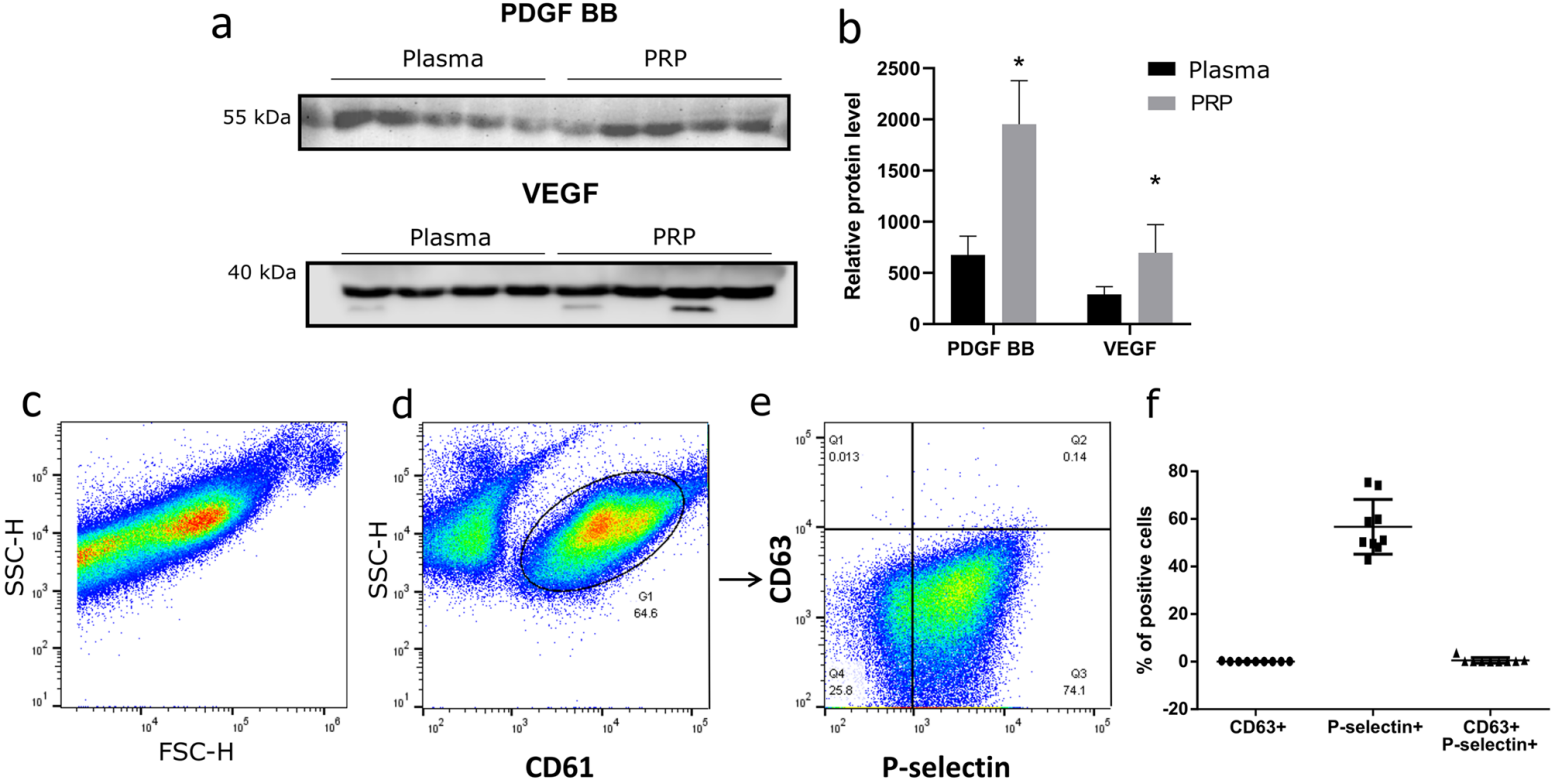
Immunochemical characterization of PRP. (**a)** Images of western blotting membranes stained with specific antibodies to PDGF-BB and VEGF, representing ordinary plasma and PRP. (**b)** relative levels of PDGF-BB and VEGF in ordinary plasma and PRP, normalized to the total protein content; *p < 0.05. (**c**, **d** and **e)** representative flow cytometry dot plots of forward *vs* side scatter (FSC *vs* SCC) and SCC *vs* CD61 gating for CD63 *vs* P-selectin, quadrant axes set according to staining control. (**f)** percentages of positively stained cells. Software: (**a)** Image Lab Software RRID: SCR_014210, (**b,f)** GraphPad Prism RRID: SCR_002798, (**c-e)** BD CellQuest Pro RRID: SCR_014489.

The samples of autologous PRP were analyzed for the content of PDGF-BB and VEGF proteins in comparison with ordinary blood plasma from the same biological samples. Western blot analysis revealed both proteins in all paired samples of PRP and ordinary plasma (Fig. 2a). Relative levels of PDGF-BB (i.e. normalized to the total protein content) were 2.8 times higher in PRP compared with ordinary plasma (*p* = 0.001; Fig. 2b). Similarly, the levels of VEGF normalized to the total protein content were 2.4 times higher in PRP compared with ordinary plasma (*p* = 0.03; Fig. 2b).

#### Platelet activation

The next step in PRP characterization was immunophenotyping with antibodies to the general platelet marker CD61 (platelet glycoprotein IIIa) and platelet activation markers CD63 (a member of tetraspanin superfamily) and P-selectin (cell adhesion molecule; Fig. 2c–f). CD61 signals allowed adequate separation of thrombocytes for subsequent counting of CD63 and P-selectin positive events confined to the main group demarcated in Fig. 2d.

The low content of CD63 positive (and, accordingly, CD63/P-selectin double-positive) platelets in PRP (< 1% in all samples, Fig. 2e) indicated the excellent preservation of non-degranulated platelets. On the other hand, high percentage of P-selectin positive particles (56.7 ± 11.5%; the lower right quadrant in Fig. 2e, f) indicated the early stages of platelet activation.

### Immunochemical characterization of the minimally manipulated cells from endometrial biopsies

**Figure 3:**
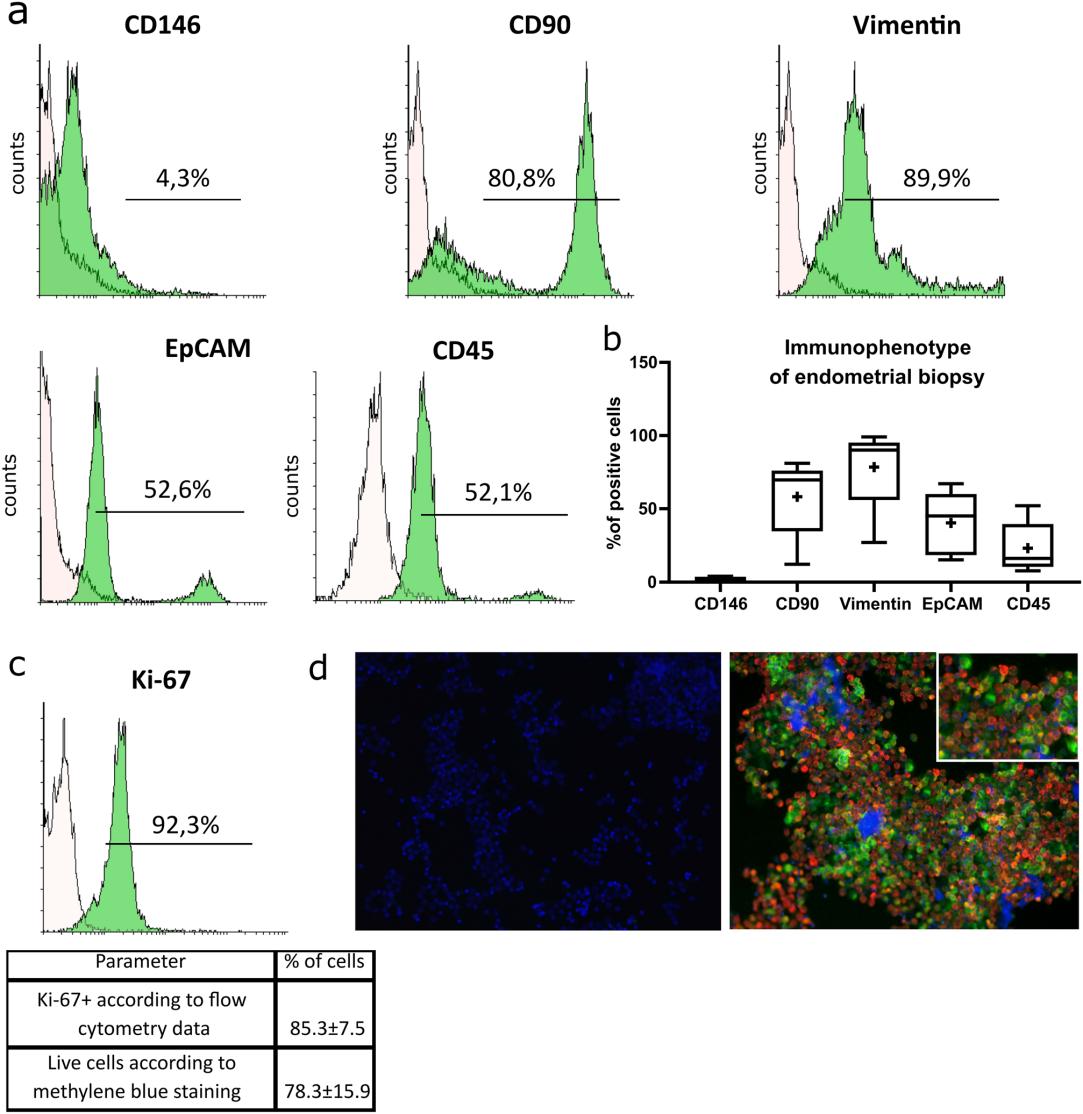
Immunochemical characterization of the minimally manipulated cell products isolated from endometrial biopsies. (**a)** representative flow cytometry histograms with indicated percentages of CD146, CD90, vimentin, EPCAM and CD45 positive events; the pale-filled curves correspond to control staining. (**b)** a boxplot of cells positivity stained by markers. **c**, cell viability evaluation by anti-Ki-67 flow cytometry and methylene blue assay. (**d)** immunocytochemistry analysis with DAPI counterstaining for cell nuclei (blue): negative control (secondary antibodies only, left image) and staining with antibodies (right image) to vimentin (red) and EPCAM (green), magnification × 200. Software: (**a,c)** Flowing Software 2.5.1 RRID: SCR_015781, B, GraphPad Prism RRID: SCR_002798.

The minimally treated cells isolated from endometrial biopsies were immunophenotyped by flow cytometry. Representation of CD146 + cells was low (2.7% on average, Fig. 3), i.e. the cultures were almost depleted of vascular elements. Instead, the cultures were positive for MSC markers CD90 (69.7%) and vimentin (91.3%), as well as the epithelial marker EPCAM (45.0%; Fig. 3a,b). Strong positivity for EPCAM and vimentin was also demonstrated immunocytochemically using fluorescence microscopy (Fig. 3d). CD45 (leukocyte common antigen) was present on 27.1% of isolated cells (Fig. 3a). Thus, the minimally manipulated cell cultures isolated from endometrial biopsies constituted heterogeneous cell populations comprising mesenchymal stromal cells, epithelial cells and leukocytes, with a minor presence of endothelial cells.

Viability of the cultures was assessed by anti-Ki67 flow cytometry and methylene blue staining (Fig. 3c). The high content of Ki-67 + cells (85.3% on average) indicated robust proliferation, although the methylene blue viability indexes were lower (78.3% on average).

### Reproductive outcomes

After the treatment, all patients entered ET cycles. The outcomes for Groups 1–3 are summarized in Table 5.

**Table 5:** Reproductive outcomes for Groups 1–3.

Indicators	Group 1 (n = 30)	Group 2 (n = 42)	Group 3 (n = 38)	p-value
ET cancellation	7 (23.3%)	6 (14.3%)	6 (15.7%)	0.77
Clinical pregnancy	6 (20%)	14 (33.3%)	12 (31.6%)	0.46
Pregnancy loss at 7–8 weeks	2 (33.3%)	4 (28.5%)	2 (16.6%)	0.65
Live birth	4 (13.3%)	9 (21.4%)	9 (23.7%)	0.59

The data are presented as raw counts and % incidence, subject to exact Fisher test.

One patient in Group 2 and one patient in Group 3 experienced spontaneous pregnancy loss at 18–19 weeks due to isthmic-cervical insufficiency. In Group 4 (n = 5) three patients successfully entered pregnancies concluded with full-term healthy deliveries.

The rates of clinical pregnancies and live births in all groups were statistically similar. At the same time, the odds of clinical pregnancy for Groups 2 and 3 were, respectively, 2 and 1.8 times higher compared with Group 1, the 95% confidence intervals (CI) for the odds ratio (OR) being [0.6, 7.3] and [0.5, 6.9], respectively. In addition, the rate of live births for Groups 2 and 3 was higher compared with Group 1, while the odds of live birth for Groups 2 and 3 were, respectively, 1.8 and 2 times higher compared with Group 1, the 95% CI for OR being [0.4, 8.7] and [0.5, 10.0]. The odds of early pregnancy loss for patients receiving conservative therapy (Group 1) were 2.4 times higher (95% CI for OR [0.1, 44.1]) compared with patients receiving injections of autologous PRP as a part of complex therapy in ART programs.

## Discussion

Successful pregnancy onset requires both the fully functional, receptive endometrium and a good quality embryo. The opportunities of influencing the embryonic factor are limited (the only means is selection), whereas the uterine factor is more manipulable. The literature contains multiple lines of evidence on the strong association of endometrial thickness with the rates of clinical pregnancy and live birth in ART programs.

First our step was estimation of patients’ endometrium. Statistically significantly higher expression of CD34 in the endometrium of patients with pregnancy reflects the pathological state of the endometrial stroma. Such a state of the stromal component, even with the unchanged glandular component of the uterine mucosa, may be an expression of immunophenotypic pathology preceding the occurrence of histologically verifiable abnormalities. It should be noted that data on increased CD34 expression in patients with various causes of infertility were also obtained by Mai et al., who assigned stromal cell pathology a leading role in the development of infertility^12^. There are no statistically significant differences in the expression of PGR in patients with an unintended pregnancy, which is inherent in an unchanged endometrium and was also found in this study in patients with an advanced pregnancy. Similar results were obtained in some studies, in which a change in the expression of PGR in the glandular component of patients with implantation disorders of various genesis was noted^13–15^.

Non-invasive intrauterine instillations with 1–2 mL autologous PRP represent a promising therapeutic option for thin endometrial infertility^16–20^. The instillations are administered at proliferative phase of the menstrual cycle followed by ET during implantation window of the same cycle (provided that endometrial thickness has reached 7 mm). Used add-on to hormone replacement cycles, such instillations reinforce (thicken) the endometrium and significantly improve the implantation and pregnancy rates in ART programs. Most settings for PRP preparation use available medical supplies (tubes, etc.) to afford about 3–4 mL of the final product. To compensate for the small volume of the product and to increase its efficacy, a number of protocols apply pre-activation of PRP by adding a mixture of thrombin powder and calcium chloride^4^. In this study, we propose an alternative strategy of injecting non-activated PRP in order to achieve degranulation of the platelets at the very moment of injection, to ensure the immediate delivery of secreted growth factors to the cells of the basal layer of endometrium.

Here we firstly describe injections of PRP into endometrium under hysteroscopic control in patients with the uterine factor infertility. We applied autologous PRP prepared in accordance with the manufacturing and quality control standards, with mandatory functional assessment of the product (platelet counts, growth factor content) immediately before the use. To maximize the curative effects of PRP, optionally with an admixture of autologous minimally manipulated cells, the medication was injected into endometrium via endoscopic needle, to the depth of 2–3 mm, under direct hysteroscopic visualization. This technique afforded a decisive improvement in many patients with refractory thin endometrium and a history of ET cancellations due to habitual endometrial insufficiency even under high doses of estrogens.

The curative effects of PRP are mediated by growth factors contained in platelet granules and released in response to activating stimuli. Platelet-derived growth factors (PDGFs) constitute a family of secreted dimeric polypeptides, which exert strong mitogenic action on cells of mesenchymal origin^21^. PDGF signaling facilitates proliferation and angiogenesis; more specifically, it promotes cytoskeletal plasticity and cell migration, while inducing extracellular matrix production and remodeling^22^. PDGFs are also produced by fibroblasts, MSCs, osteoblasts, chondrocytes, endothelial cells, monocytes/macrophages and smooth muscle cells^23^. The increased content of PDGFs (exemplified by PDGF-BB) in PRP compared with ordinary plasma triggers tissue remodeling and repair^22^. VEGFA also plays an important role in angiogenesis by regulating proliferation and migration of endothelial cells and vascular permeability. VEGFA signaling supports tissue homeostasis and promotes accelerated repair in the case of damage. VEGFA is a component of platelet α granules^24–26^. The high content of PDGF-BB and VEGF in PRP confirmed by the analysis is consistent with the observed clinical efficacy.

Platelet degranulation is triggered by specific stimuli. The process of receiving these stimuli and their interpretation by a platelet is termed 'activation'. In the context of clinical use of PRP, exact time point(s) of platelet activation are critically important. The activation status of PRP can be measured by relative counts of CD63 and P-selectin signals in the pool of CD61 positive events. CD61, also known as platelet glycoprotein IIIa, resides at the surface of platelets, both resting or activated. In a dimeric complex with CD41, it promotes platelet aggregation through interactions with plasma proteins, notably fibrinogen. By contrast, tetraspanin CD63 and P-selectin epitopes are confined within the granules — their relocation to the surface indicates degranulation. P-selectin is contained in α-granules and externalized faster than CD63. High levels of CD63–P-selectin+ platelets correspond to early stages of activation; the uniformity of this condition (observed for all PRP samples in the study) suggests that degranulation occurs upon injection (which is optimal, considering the short half-lives of growth factors).

In addition to the major part of the cohort receiving injections of unadmixed PRP or no injections at all (Groups 1–3), a small group of patients (Group 4) received injections of autologous PRP reinforced with eutopic autologous stem cells isolated from endometrial biopsies. Immunophenotyping of the minimally manipulated cell products revealed > 50% positivity for CD90 и vimentin. CD90 (which belongs to immunoglobulin superfamily of cell surface receptors) is expressed on MSCs, fibroblasts and endothelial cells^27^. Vimentin is a structural protein, the major component of intermediate filaments in cells of mesenchymal origin^28^. Apart from that, about 45% of the cells expressed EPCAM — a surface glycoprotein with Ca^2+^-independent cell adhesion molecule and transmembrane receptor functionalities. EPCAM is expressed by diverse epithelial lineages including certain populations of epithelial stem cells^29^. Identification of vimentin + EPCAM + double-positive cells by fluorescence microscopy may indicate the epithelial-mesenchymal transition characteristic of endometrial niches^30^. The viability assay was encouraging: > 70% of the cells were highly proliferative, according to Ki-67 positivity index. Thus, the product contained a highly proliferative heterogeneous cell population with a balanced content of stromal and epithelial elements. Hysteroscopic injections using this product afforded significant improvement in uterine hemodynamics confirmed by M-mode ultrasonography, which is consistent with other studies^9,11^.

The ultimate moment of truth for the infertility treatment efficacy is provided by pregnancy rates. In our setting, the estimated reproductive impact of autologous PRP injections in patients with refractory thin endometrium was positive. Both pregnancy and live birth rates in PRP-treated groups were higher compared with the control. It is important to emphasize that the patients had a history of unresponsiveness to standard therapies involving basic and add-on approaches.

## Conclusion

Overall, the study demonstrates the potential of the autologous PRP and autologous minimally manipulated endometrial cell therapies for impaired uterine hemodynamics and chronic endometrial insufficiency, as indicated by higher rates of Doppler visualization of the uterine spiral arteries and, above all, the enhanced endometrial receptivity with improved outcomes in ART programs for patients with a history of implantation failure.

## Materials and Methods

### Ethics

All procedures were performed in accordance with the Helsinki declaration and its amendments. The study was approved by Institutional Review Boards at I.M. Sechenov First Moscow State Medical University and V.I. Kulakov National Medical Research Center for Obstetrics, Gynecology and Perinatology (Doc. №10–18/11.2018). All participants signed Informed Consent for the study. Study is registered on the clinicaltrials.gov web-site (Clinical trials NCT05455151, 13/07/2022).

### Enrollment

The study was carried out in V.I. Kulakov National Medical Research Center for Obstetrics, Gynecology and Perinatology from October 2018 to March 2021. The inclusion criteria were as follows: age 18–40 years, normal karyotype, normal karyotype of the partner, regular ovulatory and menstrual cycle, endometrium < 7 mm thick as measured at implantation window, availability of ≥ 2 vitrified blastocysts of good quality, and a history of implantation failure and/or ET cancellation due to insufficient endometrial thickness. The exclusion criteria were pathospermia in partner, the use of donor gametes, premature ovarian failure, internal genital anomalies, systemic blood diseases and coagulopathy, hemoglobin < 100 g/L, platelets < 100 × 10^9^/L, and the antiplatelet/anticoagulant therapy recipient status.

The study encompassed 115 patients admitted for the treatment of thin endometrial infertility (endometrial thickness 7 mm or less at implantation window).

The participants were divided into groups by simple randomization (sequentially numbered containers). Group 1 (the control, n = 30) received electrical impulse therapy using a BTL-4000 Premium G device starting from day 5–7 of menstrual cycle for 10–12 days daily. Group 2 (n = 42) received single intraendometrial injections of autologous PRP: a 35–40 mL aliquot was injected under hysteroscopic control, via endoscopic needle, during proliferative phase (day 6–9) of menstrual cycle preceding ET. Group 3 (n = 38) received a two-step preparatory regimen spanning two consecutive menstrual cycles: at first, the patients received electrical impulse therapy identically with Group 1; over the second cycle, the patients received autologous PRP injections identically with Group 2. Group 4 (n = 5) received single intraendometrial injections of the minimally manipulated autologous endometrial cells suspended in autologous PRP: a 35–40 mL aliquot was injected under hysteroscopic control, via endoscopic needle, during proliferative phase (day 6–9) of menstrual cycle preceding ET. All patients underwent ultrasonography of pelvic organs and a Doppler ultrasound scan of uterine microcirculation. This sample size was approved by the ethics committees of the institutes. Additional information is available in Supplementary material.

### Limitation

The main limitation of the study is the small size of the group that received PRP with biopsy cells injection what is explained by the novelty of the method and the limitations of the local ethics committee.

### Histology

Pipelle biopsies for histological assessment (n = 25) were collected from patients of Group 2 at implantation window (day 5–6 after ovulation determined by luteinizing hormone tests and confirmed by ultrasound) of menstrual cycle preceding the therapy. Full information could be found in Supplementary Information.

### Immunohistochemical examination

The expression of markers such as estrogen related receptor alpha (ESRRA), progesterone receptors (PGR), LIF interleukin 6 family cytokine (LIF) and transmembrane glycoprotein CD34 was studied. Full information could be found in Supplementary Information.

### Preparation of autologous PRP

Blood samples for the procurement of autologous PRP were collected from patients of Groups 2–4 on day 5–7 of the menstrual cycle. Full information could be found in Supplementary Information.

### Western blot analysis

Western blot analysis was carried out by standard protocols, available in Supplementary Information. All uncropped membranes are available on Fig.S1 and Fig.S2.

### PRP platelet flow cytometry

Immunophenotyping of platelets in PRP was carried out in a NovoCyte Quanteon™ flow cytometer (Agilent Technologies). The samples were diluted tenfold with phosphate-buffered saline (PBS) and stained with CD61 (557291 BD Biosciences), CD63 (353011 BioLegend) and P-selectin (304910 BioLegend) at ambient temperature for 15 min. The gating strategy was aimed at counting proportions of CD63 and P-selectin positive events in CD61 + pools.

### Endometrial cell culture

For the patients of Group 4 (n = 5), endometrial aspiration biopsies were collected with intrauterine pipelles on day 6–8 of menstrual cycle (proliferative phase). Full protocol is available in Supplementary Information.

### Immunophenotyping

Isolated cells were stained for markers: CD45, EPCAM, CD146, CD90, Vimentin, Ki-67. Full protocol is available in Supplementary Information.

### Injections

All patients in Groups 2 (n = 42) and 3 (n = 38) received injections of autologous PRP on day 6–8 of menstrual cycle (proliferative phase). Group 4 (n = 5) received injections of autologous PRP mixed with autologous minimally manipulated endometrial cells isolated from aspiration biopsy specimens on the day of intervention. Full protocol is available in Supplementary Information.

### Embryo transfer

All transfers were performed in hormone replacement cycles (estradiol valerate peroral at a starting dose of 6 mg/day and micronized progesterone at a dose of 600 mg/day under dynamic ultrasound control; the maximum dose of estradiol valerate was 12 mg/day). A thawed blastocyst of good quality (according to the Gardner system^31^) was transferred to the uterine cavity under standard protocol. The post-transfer support involved 600 mg/day of micronized progesterone administered intravaginally for 2 weeks. Biochemical pregnancy was determined by measuring β-HCG serum levels on day 14 after ET; clinical pregnancy was determined by ultrasonographic visualization of gestational sac at 3 weeks after ET.

### Statistics

Associations for quantitative variables were determined by Spearman rank correlation method. Between-the-group comparisons were carried out by ANOVA/Kruskal–Wallis test for quantitative and order variables and exact Fisher test for binary and qualitative variables. The efficacy was evaluated using paired Wilcoxon test for quantitative indicators and McNemar’s binomial test for qualitative indicators. The differences were considered statistically significant at *p* < 0.05.

## Supplementary Information

Supplementary Information.

## Data Availability

The authors confirm that the data supporting the findings of this study are available within the article (and/or) its supplementary material. This study did not generate new unique reagents. The original contributions presented in the study are included in the article/supplementary files; further inquiries can be directed to the corresponding author.
