# Supplementary material for: Hysteroscopic injections of autologous endometrial cells and platelet-rich plasma in patients with thin endometrium: a pilot randomized study
Source: Sci Rep. 2023 Jan 18;13:945. doi: 10.1038/s41598-023-27982-w (PMC9849213; doi:10.1038/s41598-023-27982-w)
Supplement: Supplementary file 1 — Supplementary Information. [file 41598_2023_27982_MOESM1_ESM.docx]

**Supplementary Information**

***Enrollment***

The patients were subject to complete medical examination, with ultrasound scans of pelvic organs scheduled to implantation windows, to assess endometrial thickness and Doppler scans for uterine hemodynamics using Canon (Toshiba) Aplio 500 system with a transvaginal sensor. The scans were scheduled to implantation windows on day 5–6 after ovulations determined by luteinizing hormone (LH) surge in urine and confirmed by ultrasound scans.

***Histology***

The material was fixed in 10% neutral formalin, dehydrated, paraffin-embedded and sectioned. The 4-5 µm serial sections were stained with H&E and examined microscopically at x50-x400 magnifications to assess the functional condition of stromal and glandular elements and vascularization status consistently with the phase.

***Immunohistochemical examination***

Immunohistochemical reactions were carried out on paraffin sections with a thickness of 4 microns, located on glasses coated with L-lysine in automatic immunohistochemical stainer. The expression of markers such as estrogen receptor α (ERΑα), progesterone receptors (PgR), leukemia-inhibitory factor (LIF) and transmembrane glycoprotein CD34 was studied. Evaluation of the expression of the markers was carried out by a semi-quantitative method, taking into account the intensity and percentage of positively stained cells with the calculation of the H-score indicator, which was calculated using the H-score formula = *3a+2b+1c*, where *a* is a pronounced intensity of expression, *b* is a moderate intensity of expression, *c* is a weak intensity of expression. The assessment was carried out if at least 1% of the cells showed expression of the corresponding marker. With an H-score from 0 to 100, expression was considered weak, from 101 to 200 – moderate, from 201 to 300 - strong ^1,2^. CD34 expression was also evaluated by a semi-quantitative method taking into account the percentage of stroma cells expressing protein to the total number of stroma cells, while the result was expressed in points that corresponded to the following parameters: 0 points – lack of expression or expression in <1% of stroma cells, 1 point – expression in 1-5% of stroma cells, 2 points – expression in 5-10%, 3 points - expression in 10-30%, 4 points - expression in 30-60%, 5 points – expression in >60% ^3^.

***Preparation of autologous PRP***

Before the procedure, the patients underwent blood testing and were consulted by a transfusiologist for contraindications to confirm eligibility. The procedure was carried out in the Department of transfusion immunology and processing of blood components of the V.I. Kulakov Research Center as follows: (1) collection of the blood (400±50 mL, corresponding to 10% of circulating blood volume) into triple blood bags with СPDA (Ravimed, Poland); (2) centrifugation in a blood bag centrifuge (Beckman Coulter) at 1 971 g for 8 min at 22º С; (3) collection of autologous red blood cells for reinfusion; (4) additional centrifugation of the bag with plasma and platelets at 5 130 g for 10 min at 22 ºС; (5) the upper layer was removed with a manual plasma extractor and the remaining PRP was transferred to a platelet incubator (Lmb Technologie GmbH, Germany) for up to 5 day storage at 22–24 ºС. The procedure yielded 35–40 mL of autologous PRP containing a total of 0.6–0.7×10^11^ platelets, delivered to the operating room on the day of injection.

***Western blot analysis***

To obtain ordinary plasma (no platelets), PRP was centrifuged at 2000 g for 10 min at +4 ºC and the supernatant was collected. Aliquots of plasma and PRP were diluted 10-fold and mixed with Laemmli sample buffer (Bio-Rad). The proteins were separated by 10–12.5% SDS-PAGE and transferred to nitrocellulose membranes (Millipore) at 0.35 A for 1 h. The membranes were blocked in Tris-buffered saline with 5% w/v nonfat dry milk at ambient temperature for 1 h. Primary antibodies (PDGF-BB, ab9704 Abcam, and VEGF, ab46154 Abcam) were applied at +4 °С for 18 h and secondary antibodies were applied at ambient temperature for 1 h. The bands were developed with ECL substrate (Invitrogen) with subsequent detection in a ChemiDoc imaging system (Bio-Rad) and analysis of signal intensity in ImageLab software (Bio-Rad). All uncropped membranes are available on Fig.S1 and Fig.S2. Relative contents of PDGF-BB and VEGF were determined through normalization of the corresponding signal intensities by total protein concentrations measured with Bradford assay using a SmartSpec Plus spectrophotometer (Bio-Rad) and serial dilutions of BSA (0.2 to 2 mg per sample) to build a standard curve for the optical density at 595 nm.


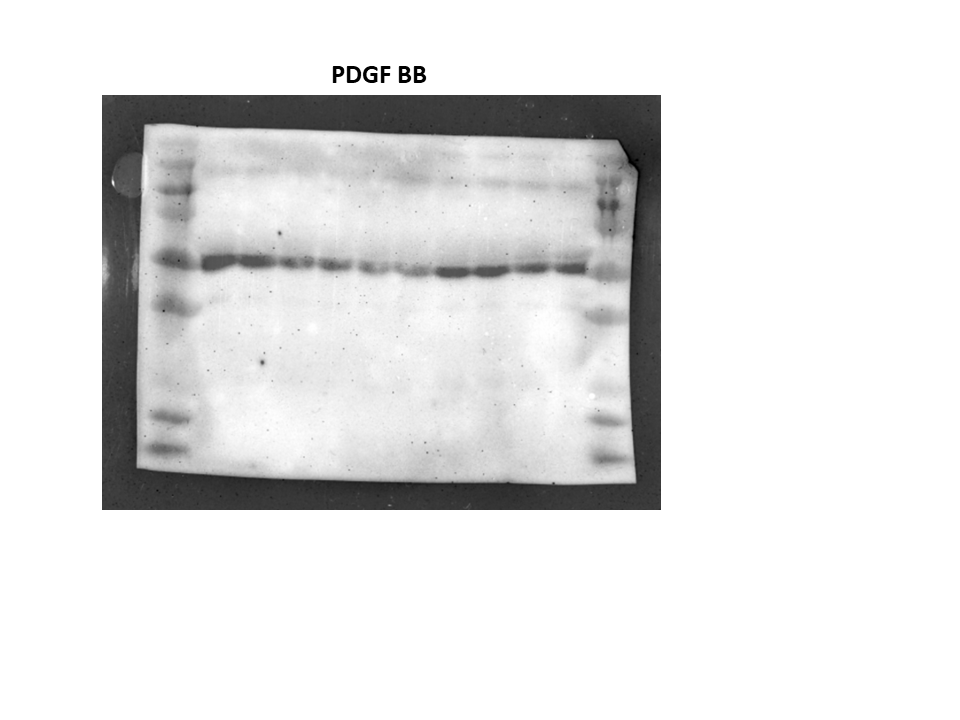


Fig.S1. Uncropped membrane stained with antibodies to PDGF BB.


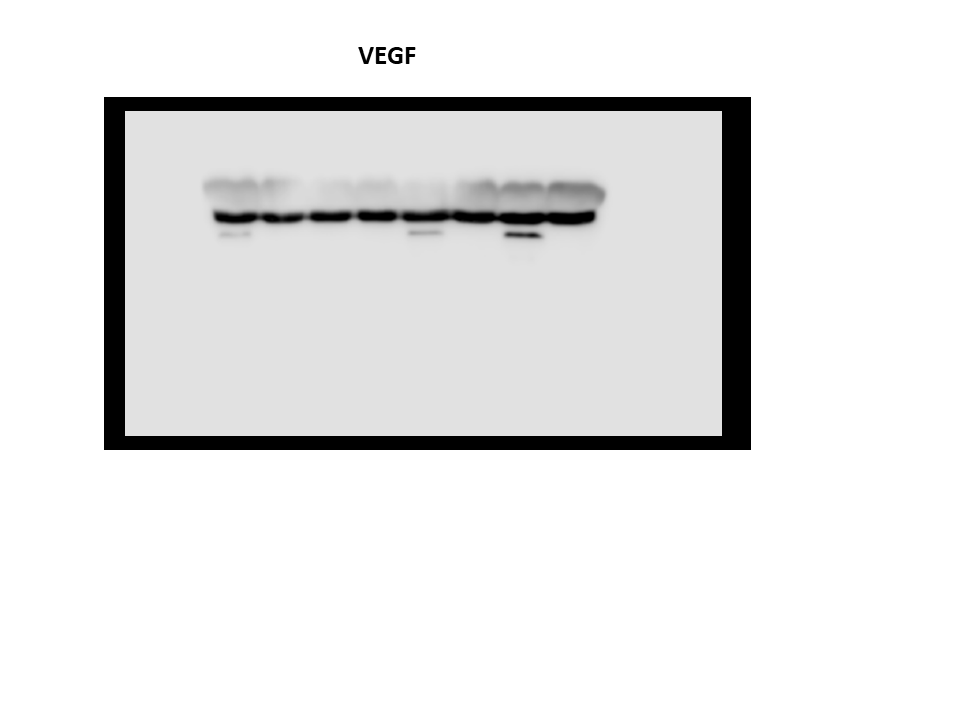


Fig.S2. Uncropped membrane stained with antibodies to VEGF.

***Endometrial cell culture***

The samples were immediately placed in a 15 mL tube with F12 (PanEco) with 1% penicillin-streptomycin for transportation. In a sterile tissue culture box, the material was transferred to a Petri dish, washed with Hanks balanced salt solution (HBSS), and minced with fine scissors. The material was passed through a G21 syringe needle (with a 1/10 v/v withheld for immunophenotyping), washed twice with HBSS, diluted with autologous PRP, and delivered to the operating room. Viability of the cultures was assessed by methylene blue staining in automatic cell counter TC20 (Bio-Rad).

***Immunophenotyping***

An aliquot of the minced endometrial tissue was transferred to a Petri dish containing 5 mL of 0.05% trypsin in HBSS and incubated at ambient temperature for 10 min. The digestion was terminated by adding equal volume of 2% FBS in RPMI (Wash Buffer). The sample was centrifuged at 500 g for 10 min and the supernatant was discarded. The pellet was mixed with 5 mL Wash Buffer, passed through a 100 µm strainer, and centrifuged again at 500 g for 10 min. The cells were resuspended in 200 µL PBS and mixed with 800 µL of red blood cell lysis buffer. The mixture was incubated on ice for 10 min and centrifuged at 500 g for 10 min; the cells were resuspended in PBS, counted and diluted on the basis of 10^5^ cells per analysis. Immunophenotyping was carried out according to conventional protocols. To stain for internal markers vimentin MA1-19656 and Ki-67 130-100-290, the cells were fixed and permeabilized using Inside Stain kit (Miltenyi Biotec) as described by manufacturer. To stain for surface markers, the cells were incubated in 100 µL PBS with 1% BSA and the antibodies (CD45 A07785, EpCAM MA1-10197, CD146 130-092-851, CD90 130-117-388) for 10 min on ice, washed, resuspended in 500 µL of PBS and analyzed on a BD FACSCalibur flow cytometer (BD Biosciences).

For immunocytochemistry, the cells were plated on coverslips (Fisher Scientific; 10^5^ cells per coverslip) and fixed in 2% paraformaldehyde at ambient temperature for 10 min, washed with PBS, blocked in 1% BSA for 30 min, incubated with antibodies to vimentin (ab8978) and EpCAM (sc-23788) at 4 °C for 24 h, washed again, incubated with FITC- and PE-conjugated secondary antibodies at ambient temperature for 1 h, counterstained with DAPI (0.004 mg/mL at 37 °C for 10 min) and embedded in Aqua-Poly Mount (Polysciences). The fluorescence images were taken using Leica DM 4000B microscope with LAS AF v.3.1.0 software (Leica Microsystems, Wetzlar, Germany).

***Injections***

All injections were performed identically, in operating facilities under intravenous anesthesia; the 35‑40 mL injection volume was administered with a 0.6/1.16 mm endoscopic needle to the depth of 2‑3 mm under hysteroscopic control (Fig.1a). The entire procedure took 15‑20 min and involved 6‑8 insertions of the needle into altered areas of endometrial tissue. After 4-hour postoperative observation in a day hospital, the patients were discharged in satisfactory condition. The patients were prescribed mechanical contraception and no ETs in the ongoing menstrual cycles were carried out. None of the patients manifested allergic reactions or infectious complications following the intervention.

**References**

1. Ma, H. *et al.* Quantitative measures of estrogen receptor expression in relation to breast cancer-specific mortality risk among white women and black women. *Breast Cancer Res.* **15**, R90 (2013).

2. Margioula-Siarkou, C. *et al.* LIF and LIF-R expression in the endometrium of fertile and infertile women: A prospective observational case-control study. *Mol. Med. Rep.* **13**, 4721–4728 (2016).

3. Mai, K. T., Teo, I., Al Moghrabi, H., Marginean, E. C. & Veinot, J. P. Calretinin and CD34 immunoreactivity of the endometrial stroma in normal endometrium and change of the immunoreactivity in dysfunctional uterine bleeding with evidence of ‘disordered endometrial stroma’. *Pathology* **40**, 493–499 (2008).
